# MRI of the Scrotum and Penis: Current Applications and Clinical Relevance

**DOI:** 10.3390/diagnostics15243134

**Published:** 2025-12-09

**Authors:** Bartosz Regent, Karolina Nowak, Katarzyna Skrobisz, Marcin Matuszewski, Michał Studniarek

**Affiliations:** 1 Department of Radiology, Medical University of Gdańsk, 80-210 Gdańsk, Polandmichal.studniarek@gumed.edu.pl (M.S.); 2 Department of Urology, Medical University of Gdańsk, 80-210 Gdańsk, Poland

**Keywords:** magnetic resonance imaging, scrotum, penis, testicular tumor, penile cancer, infertility, Peyronie’s disease, priapism, multiparametric MRI

## Abstract

**Background:** Magnetic resonance imaging (MRI) plays an increasingly important role in the evaluation of scrotal and penile disorders, complementing ultrasonography in cases where findings are equivocal or complex. With its superior soft-tissue contrast, multiplanar capability, and advanced functional sequences, MRI provides unparalleled anatomic and tissue characterization across a wide range of male genital pathologies. **Summary:** This review summarizes current clinical applications of MRI in scrotal and penile imaging and discusses its diagnostic value, protocol optimization, and interpretive features. In scrotal pathology, MRI accurately differentiates torsion, trauma, infection, and neoplasms, aiding in the distinction between benign and malignant testicular lesions and supporting testis-sparing management. Quantitative diffusion and perfusion metrics further refine lesion characterization. In andrology, MRI biomarkers such as apparent diffusion coefficient (ADC), magnetization transfer ratio (MTR), and proton spectroscopy serve as promising non-invasive indicators of spermatogenic activity in male infertility. In penile imaging, MRI enables precise local staging of carcinoma, assessment of plaque morphology and activity in Peyronie’s disease, evaluation of tissue viability in priapism, and detection of prosthesis-related complications. **Conclusions:** MRI has become an essential problem-solving tool in the assessment of scrotal and penile diseases, enhancing diagnostic confidence and surgical planning. Future directions include protocol standardization, quantitative parameter validation, and the integration of radiomics and artificial intelligence to improve reproducibility and clinical impact.

## 1. Introduction

Magnetic resonance imaging (MRI) is an increasingly important modality for the comprehensive evaluation of scrotal and penile pathologies. While ultrasonography (US) remains the first-line imaging tool due to its wide availability, low cost, and high diagnostic performance for many urological indications, it has inherent limitations—including operator dependency, restricted field of view, and challenges in tissue characterization. In complex or equivocal cases, MRI serves as a powerful problem-solving tool, offering superior soft tissue contrast, multiplanar imaging capabilities, and functional data through advanced sequences such as diffusion-weighted imaging (DWI) and dynamic contrast-enhanced (DCE) imaging [1,2,3].

Despite its diagnostic potential, scrotal and penile MRI remains underutilized in routine clinical practice, partly due to limited training exposure among radiologists. However, recent advances in MRI techniques and emerging evidence have solidified its role in differentiating benign from malignant lesions, staging neoplasms, characterizing inflammatory and traumatic conditions, and guiding surgical or conservative management strategies. Multiparametric MRI (mpMRI) protocols now enable accurate assessment of both anatomical and functional features, enhancing diagnostic confidence across a wide spectrum of pathologies [4,5,6].

### 1.1. Materials and Methods

This article is a narrative review synthesizing current evidence on the clinical applications, technical considerations, and diagnostic value of magnetic resonance imaging in the evaluation of scrotal and penile pathology. A structured search of the literature was performed in PubMed/MEDLINE, Scopus, and Google Scholar databases, covering publications from January 2000 to January 2025. The search strategy included the following keyword combinations: “scrotal MRI”, “penile MRI”, “testicular tumor MRI”, “penile cancer staging”, “male infertility MRI”, “priapism MRI”, and “Peyronie’s disease MRI”.

Articles were screened based on relevance to the imaging evaluation of the scrotum and penis. Priority was given to:

1. Peer-reviewed original clinical studies,
2. Meta-analyses and systematic reviews,
3. Guidelines or expert consensus statements, particularly from the European Society of Urogenital Radiology (ESUR) and the European Association of Urology (EAU),
4. High-quality pictorial reviews and technical imaging discussions.

Eligible studies included peer-reviewed human studies, adult and pediatric populations, MRI-based research focused on scrotal or penile pathology, and English-language publications with accessible full text. Case reports were included selectively when they illustrated key or rare imaging features not otherwise documented in larger studies.

Exclusion criteria included non-MRI-based studies, conference abstracts without full text, case reports without substantive imaging methodology, non-English papers, and studies lacking clear diagnostic, technical, or clinical outcomes.

When multiple studies addressed similar topics, prioritization was based on sample size, methodological quality, recency, and alignment with current guidelines.

All figures presented in this manuscript originate from the clinical imaging archive of the Department of Radiology, Medical University of Gdańsk. These images were obtained as part of routine patient care, anonymized prior to use, and no copyrighted materials were reproduced.

As this article is a review and does not involve new data collection from human participants, Institutional Review Board approval was not required.

During the preparation of this manuscript, the authors used Grammarly (version 1.2.95) for English language correction and refinement of grammar and syntax. No generative AI tools were used to create, analyze, interpret, or revise scientific content. All substantive content, the literature synthesis, and clinical interpretations were performed directly by the authors, who take full responsibility for the accuracy, integrity, and originality of the work.

### 1.2. Imaging Technique

High-quality MRI of the penis and scrotum requires meticulous technique to maximize diagnostic value and anatomic visualization. The examination is generally performed with the patient in the supine and feet-first position, using either a 1.5 T or 3.0 T scanner. To stabilize and straighten the penis, it is dorsiflexed and taped to the lower abdominal wall, while the scrotum is elevated with a towel placed between the thighs. This positioning minimizes motion and curvature, facilitates clear delineation of penile structures, and ensures both testes are symmetrically oriented within the coil’s field of view [3,7,8].

A multichannel phased-array surface coil is positioned over the lower abdomen and pelvis to enhance spatial resolution. MRI of the penis and scrotum typically includes high-resolution T2-weighted turbo spin echo (TSE) sequences in three orthogonal planes (axial, coronal, and sagittal), with a small field of view (FOV; ~16 cm) and thin slices (3–4 mm). Fat-suppressed T2-weighted sequences are also acquired, particularly to assess for fluid collections, edema, or inflammation [4,9,10].

T1-weighted images, including in-phase and out-of-phase sequences, may be used to assess for fat-containing lesions and characterize blood products via T2* effects. DWI is increasingly used for characterization of infections, tumors, and undescended testes, while gadolinium-enhanced sequences (3D spoiled gradient echo) assist in assessing enhancement patterns of penile and scrotal lesions [7,9,11].

In the evaluation of penile prostheses, MRI is usually safe, except in patients with certain older devices like the OmniPhase or DuraPhase implants, which may be subject to magnetic deflection. In selected cases, an artificial erection may be induced using intracavernosal prostaglandin E1 injection, though this is not routinely practiced due to workflow limitations and contraindications in patients predisposed to priapism [2,12].

## 2. Normal Anatomy

### 2.1. Penis

**Figure 1:**
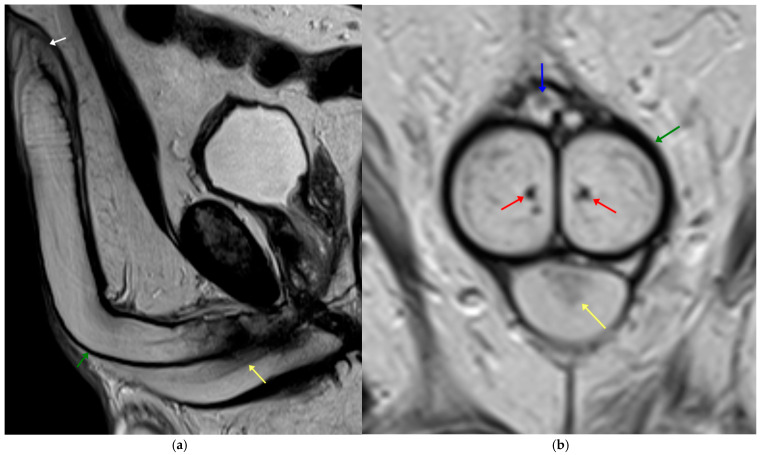
Normal penile anatomy on T2-weighted MRI. (**a**) Sagittal and (**b**) coronal images. Green arrows indicate the tunica albuginea; yellow arrows denote the urethra centrally located within the corpus spongiosum. The glans penis is marked with a white arrow. Red arrows identify the cavernosal arteries, and the blue arrow highlights the superficial dorsal vein.

The penis is composed of three erectile bodies: two corpora cavernosa and the corpus spongiosum. These structures appear with high signal intensity on T2-weighted MRI and intermediate-to-low signal on T1-weighted imaging (Figure 1). The corpora cavernosa are separated by a porous midline septum that permits blood flow between the two sides. All three erectile bodies are encased by the tunica albuginea, a dense fibrous sheath that appears uniformly low in signal on MRI, with maximal contrast to the surrounding corpora on T2 sequences. Superficial to the tunica lies Buck’s fascia, another fibrous layer that is sometimes distinguishable on imaging, and more superficially, the thin Dartos fascia, loose connective tissue, and skin. The corpus spongiosum surrounds the urethra, expands proximally into the bulb (encircled by the bulbospongiosus muscle), and distally continues as the glans penis, where the tunica albuginea fuses with subepithelial connective tissue. Vascular supply derives from the common penile artery, a branch of the internal pudendal artery, which gives rise to cavernosal, dorsal, and bulbourethral branches, while venous drainage occurs mainly through the superficial and deep dorsal veins located in the midline between Buck’s fascia and the tunica albuginea [12,13,14].

### 2.2. Scrotum

**Figure 2:**
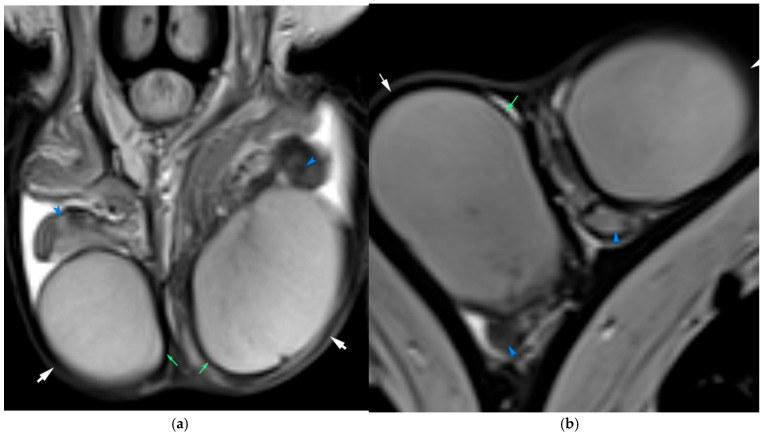
Normal scrotal anatomy on T2-weighted MRI. (**a**) Coronal and (**b**) sagittal images. Green arrows indicate the tunica albuginea; white arrows outline the scrotal wall. The epididymides are depicted by blue arrowheads. A small bilateral hydrocele is present, regarded as a normal incidental finding.

The scrotum comprises skin, subcutaneous tissue, and multiple fascial layers, including the tunica vaginalis—a two-layered serous membrane derived from the peritoneum. The visceral layer envelops the testes and epididymis, while the parietal layer lines the inner scrotal wall. The tunica vaginalis forms a potential space that may accumulate fluid, as in hydrocele. On MRI, the scrotal wall typically appears hypointense on both T1- and T2-weighted sequences. The testes are ovoid, homogeneous structures that appear hyperintense on T2-weighted images and iso- to hypointense on T1-weighted sequences (Figure 2). The tunica albuginea surrounding the testes is again hypointense on all sequences. The epididymis is iso- to hypointense relative to the testis and enhances following gadolinium administration. MRI can depict the mediastinum testis, testicular septa, and the components of the spermatic cord, including the vas deferens, testicular vessels, lymphatics, and nerves. These are best visualized on T2-weighted imaging, where the fascial layers and vascular anatomy can be delineated clearly, especially in axial and coronal planes [3,5,15,16].

## 3. Scrotal Pathology

### 3.1. Torsion and Trauma

According to the EAU Guidelines, the diagnosis and management of suspected testicular torsion are primarily based on clinical findings alone, as any delay to surgery may compromise testicular viability. When imaging is required, Doppler ultrasonography remains the first-line modality, used only when clinical uncertainty exists and when it does not prolong time to surgical exploration [17,18].

**Figure 3:**
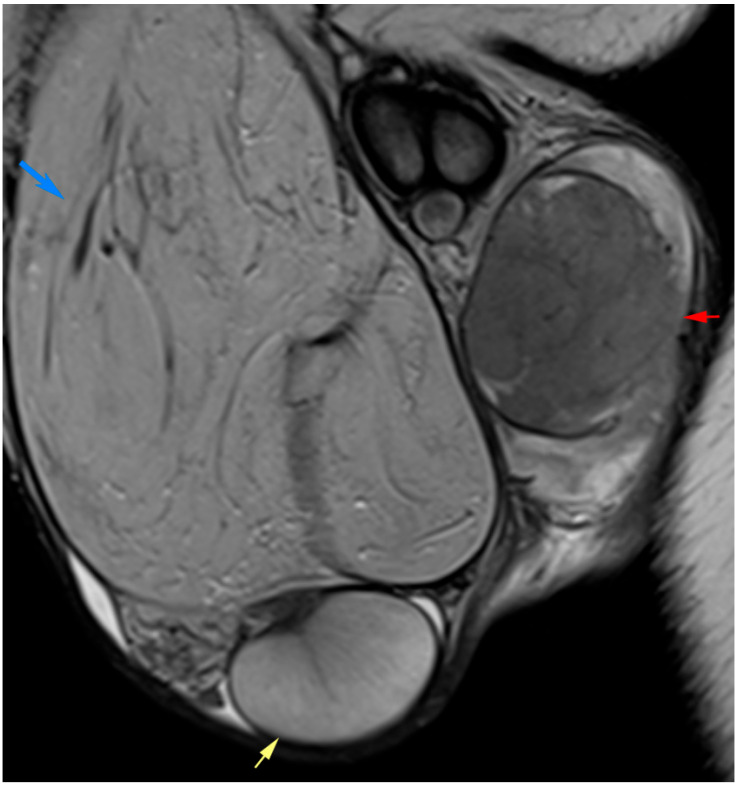
Testicular torsion. Coronal T2-weighted image demonstrates the left testis (red arrow) with irregular margins and markedly hypointense signal consistent with necrosis, in contrast to the normal right testis (yellow arrow). A large right-sided scrotal hernia is also present (blue arrow).

Although not recommended in emergency algorithms, several publications report that MRI may be useful in highly selected, equivocal cases when ultrasonography is inconclusive and when its use does not delay surgery [19,20,21]. Typical MRI findings include an enlarged and swollen testis with diminished or absent internal enhancement on contrast-enhanced images, indicative of compromised blood supply. A characteristic MR finding is the “whirlpool sign,” reflecting the twisted appearance of the spermatic cord, readily identifiable on axial or sagittal T2-weighted images and further reinforced by post-contrast imaging sequences. Furthermore, DWI may show markedly restricted diffusion, a hallmark of acute testicular ischemia [5,20,21]. In a prospective study, DCE-MRI demonstrated 100% sensitivity for diagnosing complete torsion, while T2-weighted and T2*-weighted imaging provided 100% accuracy for detecting necrosis (Figure 3). These sequences can assess testicular viability even when Doppler US is technically limited or non-diagnostic. Moreover, MRI has been suggested for postoperative evaluation of testicular perfusion following detorsion, particularly in cases of uncertain viability [20].

The “split sign” is an MRI finding recently described as the imaging correlate of the bell clapper deformity (BCD)—a congenital anomaly that predisposes to testicular torsion by allowing increased mobility of the testis within the tunica vaginalis. This sign is visualized as a hyperintense T2-weighted signal interposed between the posterior aspect of the epididymis and the scrotal wall, indicating the presence of a posteriorly placed tunica vaginalis that separates the epididymis from its usual posterior attachment. This altered anatomical configuration is characteristic of the bell clapper deformity, in which the tunica vaginalis completely encircles the testis and epididymis, enabling their free rotation [22].

Scrotal trauma may result from blunt force, penetrating injuries, or iatrogenic causes, with potential consequences including testicular hematoma, hematocele, fracture, or rupture. Although US can detect hematocele and disruption of testicular architecture, its sensitivity is limited in evaluating tunica albuginea integrity or differentiating hematomas from neoplasms, particularly in cases lacking a clear traumatic history [16,23,24]. MRI offers accurate tissue characterization, with T1-weighted imaging demonstrating hyperintensity in acute hemorrhage and T2-weighted imaging revealing parenchymal heterogeneity and tunical disruption. A key sign of testicular rupture is the discontinuity of the low-signal tunica albuginea on T2-weighted sequences [16,25]. In cases of suspected testicular hematoma with inconclusive sonography, MRI can confidently distinguish blood products from malignancy based on signal characteristics and absence of enhancement, thereby potentially avoiding unnecessary orchiectomy [15,23]. MRI has been shown to reach 100% diagnostic accuracy in trauma cases involving suspected rupture or hematoma when US was equivocal [16].

Segmental testicular infarction, which may occur following trauma, surgery, or torsion-related vascular compromise, is characteristic radiologic finding that appears on MRI as a wedge-shaped or sectoral area of low signal intensity on T2-weighted images. This non-enhancing region typically demonstrates diffusion restriction on DWI and low ADC values, distinguishing it from neoplastic or inflammatory process [26].

### 3.2. Inflammation and Infection

MRI findings in acute orchitis typically include diffuse or patchy testicular enlargement with low signal intensity on T1-weighted images and high signal intensity on T2-weighted sequences. Concomitant epididymal thickening and increased signal intensity, as well as scrotal wall thickening, are often present. Post-contrast imaging frequently reveals diffuse enhancement reflecting hyperemia and inflammation. DWI may show restricted diffusion, although this is not specific [16].

One of MRI’s most critical roles in inflammatory pathology is the differentiation between orchitis and testicular neoplasms. In the subacute or chronic phase, focal orchitis may simulate a tumor on ultrasound. However, MRI can show lack of well-defined focal masses, absence of internal necrosis or hemorrhage, and no aggressive features such as tunica albuginea invasion or peritesticular extension. Dynamic contrast-enhanced MRI and DWI can further aid differentiation, although overlap remains. In equivocal cases, MRI follow-up after antibiotic therapy may help avoid unwarranted surgery [3,27].

MRI is valuable in identifying abscess formation complicating epididymo-orchitis, which may be underappreciated on ultrasound. T2-weighted images can demonstrate well-defined hyperintense collections, while post-contrast sequences show peripheral rim enhancement. Intrascrotal fistulous tracts and extension into adjacent tissues which may be difficult to detect on sonography are well demonstrated on T2-weighted MRI, aiding surgical planning and drainage decisions [16,23].

Although rare, Fournier’s gangrene represents a urological emergency with high morbidity. MRI plays an important role in early diagnosis and surgical planning by accurately mapping the fascial spread of infection, identifying necrotic tissue, and evaluating extrascrotal extension. T2-weighted sequences are particularly effective in delineating fluid collections and soft-tissue edema, while post-contrast sequences reveal the extent of non-perfused necrotic tissues [16,28].

Table 1 provides a summary of MRI characteristics for acute scrotal conditions.

### 3.3. Scrotal Masses

According to both the EAU and the AUA, evaluation of a suspected testicular mass should begin with a focused history and physical examination, followed by scrotal ultrasonography as the first-line imaging modality, given its high sensitivity and specificity for detecting and characterizing intratesticular lesions. Both societies also recommend obtaining serum tumor markers: α-fetoprotein (AFP), β-human chorionic gonadotropin (β-hCG), and lactate dehydrogenase (LDH) in all patients with suspected testicular cancer, as they contribute to staging and risk stratification, although normal values do not exclude malignancy [29,30].

MRI has increasingly established its role as a second-line imaging modality in the evaluation of scrotal masses, particularly in sonographically indeterminate cases. While ultrasonography remains the initial and primary tool due to its wide availability, low cost, and real-time capability, it is limited in specificity and tissue characterization, especially for deep-seated, complex, or isoechoic lesions. In contrast, MRI offers high soft-tissue resolution, multiplanar capability, and functional imaging sequences, which together provide additional diagnostic value that may be helpful in management decisions, including the avoidance of unnecessary orchiectomy or guiding testis-sparing surgery (TSS) [5,31,32,33].

The primary objective in the assessment of a scrotal mass is determining whether the lesion is intratesticular or extratesticular. This distinction is crucial because over 90% of solid intratesticular masses are malignant, whereas the majority of extratesticular lesions are benign [7,34]. MRI reliably delineates anatomical boundaries and lesion origin using high-resolution T2-weighted imaging and fat-suppressed T1-weighted post-contrast sequences. The tunica albuginea, appearing as a low-signal-intensity band on both T1- and T2-weighted images, serves as a landmark to assess whether the lesion arises from within or outside the testicular parenchyma [35].

#### 3.3.1. Intratesticular Masses

Beyond localization, MRI enables characterization of tissue composition. For instance, simple cysts appear hypointense on T1-weighted images and hyperintense on T2-weighted images, whereas hemorrhagic, fibrotic, or fat-containing lesions exhibit more complex signal patterns. Functional imaging with DWI and DCE MRI further refines differentiation. Malignant lesions typically demonstrate restricted diffusion with low ADC values (<0.9 × 10^−3^ mm^2^/s), and intense, rapid, heterogeneous enhancement with contrast washout, whereas benign lesions tend to show higher ADC values and type I or II enhancement curves [27,31,36].

Several studies have validated the diagnostic accuracy of multiparametric MRI in this setting. In one prospective study, mpMRI achieved 94% sensitivity and 77% specificity in distinguishing malignant from benign testicular lesions using combined morphological, diffusion, and enhancement features [37]. Such diagnostic confidence is essential when evaluating incidentally detected nonpalpable testicular masses, which are increasingly encountered due to widespread scrotal US and may represent benign lesions such as Leydig cell tumors or epidermoid cysts [5,38].

**Figure 4:**
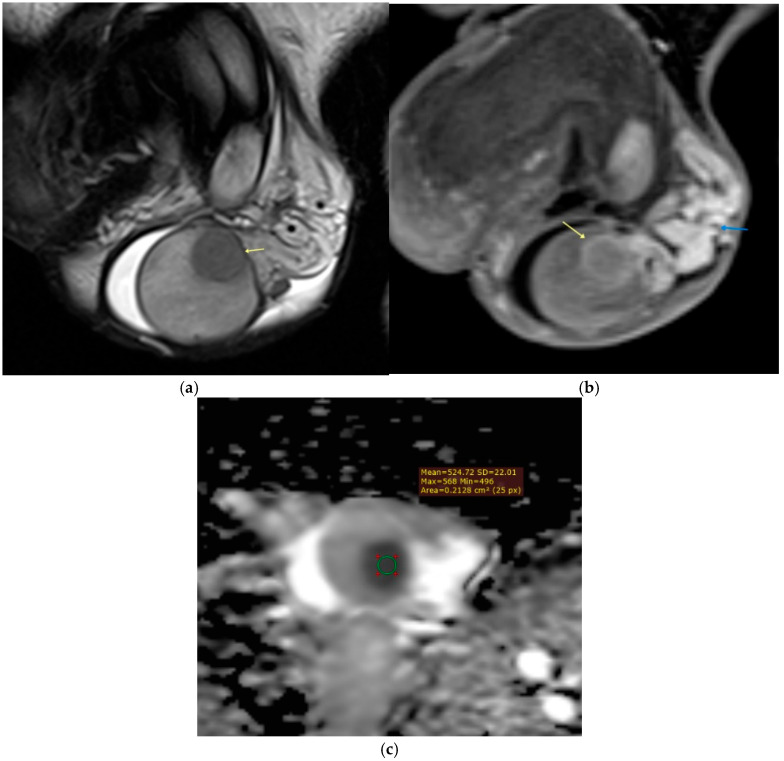
Seminoma of the left testis. (**a**) Coronal T1-weighted image depicts a well-circumscribed, homogeneous, hypointense intratesticular mass (yellow arrow). (**b**) Coronal post-contrast T1-weighted image shows homogeneous enhancement of the lesion; note the dilated, tortuous pampiniform veins consistent with varicocele (blue arrow). (**c**) Corresponding ADC map demonstrates pronounced diffusion restriction within the tumor, with an ADC value of approximately 0.5 × 10^−3^ mm^2^/s.

**Figure 5:**
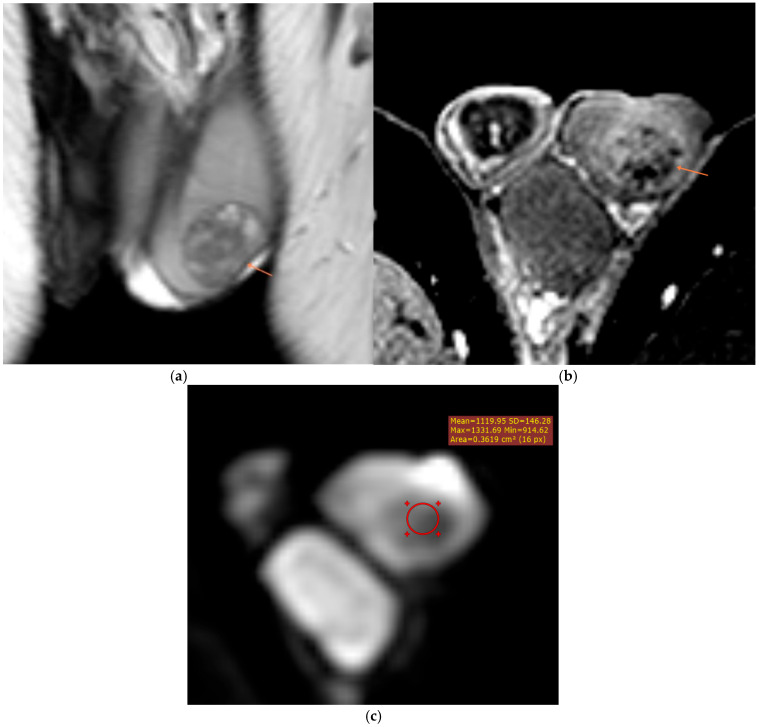
Mixed germ cell tumor of the left testis. (**a**) Coronal T2-weighted image demonstrates an irregular intratesticular mass (orange arrow). (**b**) Axial post-contrast T1-weighted image shows heterogeneous enhancement with avascular hypointense regions representing necrosis. (**c**) ADC map demonstrates mild diffusion restriction within the tumor, with an ADC value of approximately 1.1 × 10^−3^ mm^2^/s.

MRI contributes to the evaluation of testicular germ cell tumors (TGCTs), which represent the most common solid malignancies in men aged 15–40 years. Seminomas, which comprise 50–60% of TGCTs, typically present as well-defined, homogeneous, T2-hypointense masses with internal fibrous septa that show contrast enhancement (Figure 4). In contrast, nonseminomatous germ cell tumors (NSGCTs) tend to be larger and more heterogeneous, often containing hemorrhagic, necrotic, or cystic components that appear hyperintense on T2 and may show peripheral or heterogeneous enhancement [5,35,39] (Figure 5).

DWI has been proposed as a potential biomarker for distinguishing seminomas from NSGCTs. ADC values are generally lower in seminomas due to their cellular homogeneity, whereas NSGCTs demonstrate relatively higher ADC values reflecting their heterogeneous composition. These imaging biomarkers, combined with clinical data and serum tumor markers, may assist in guiding initial management, especially when orchiectomy is delayed in cases of life-threatening metastases or bilateral tumors [8,40].

MRI is also valuable for local staging. Accurate assessment of tumor extension into the rete testis, tunica albuginea, or epididymis is essential for surgical planning and may impact prognosis. The visualization of testicular capsule integrity and surrounding soft tissues is enhanced by high-resolution T2-weighted and contrast-enhanced T1-weighted imaging, helping radiologists identify candidates for TSS [1].

**Figure 6:**
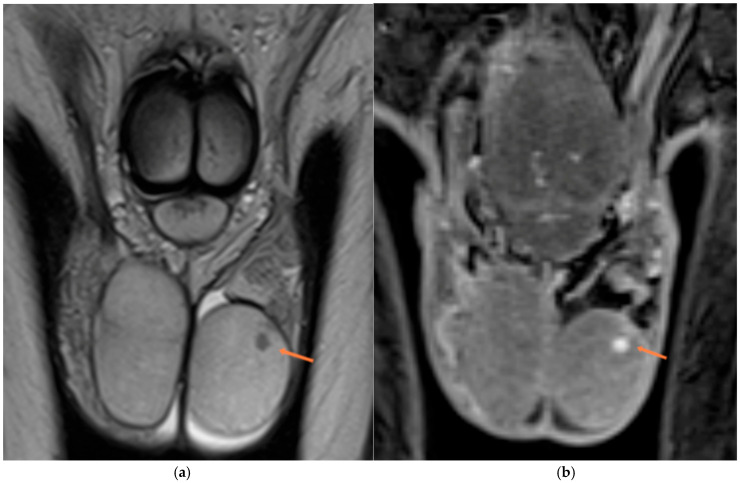
Leydig cell tumor (orange arrows). (**a**) Coronal T2-weighted image shows a small, well-defined hypointense intratesticular lesion. (**b**) Corresponding contrast-enhanced T1-weighted image demonstrates intense, homogeneous enhancement of the lesion.

While TGCTs represent approximately 95% of testicular malignancies, a small subset of solid intratesticular tumors are sex cord-stromal tumors (SCSTs), most commonly Leydig cell tumors (LCTs). Distinguishing these entities preoperatively is important, particularly in patients eligible for testis-sparing surgery or active surveillance. MRI contributes valuable information in differentiating GCTs from SCSTs. Leydig cell tumors usually present as small, well-defined, hypointense to mildly hypointense lesions on T2-weighted images, with strong, early enhancement and less diffusion restriction compared to GCTs (Figure 6). Their bilateral or multifocal nature, in combination with normal serum tumor markers, can suggest a benign stromal origin. Recognition of these imaging features helps avoid unnecessary orchiectomy and enables a more conservative surgical approach when clinically appropriate [41]. MRI features of testicular tumors are presented in Table 2.

Radiomics has recently emerged as a promising adjunct in scrotal MRI, offering quantitative tools for characterizing testicular lesions beyond conventional image interpretation. Studies applying radiomics to multiparametric MRI—using T2-weighted, DWI, ADC, and contrast-enhanced sequences show that texture-based features can differentiate benign from malignant intratesticular masses and even assist in subclassifying tumor histology [42,43,44,45]. Machine-learning models such as logistic regression and XGBoost have achieved high diagnostic performance (AUC up to 0.93), particularly when radiomics features are combined with clinical data [42,45]. Volumetric ADC metrics and fractional anisotropy (FA) histogram parameters derived from diffusion tensor imaging further enhance detection of microscopic abnormalities, including germ cell neoplasia in situ [44].

Epidermoid cysts, though uncommon, constitute a clinically significant entity due to their benign nature and the potential for organ-preserving treatment strategies. Their imaging hallmark on MRI includes a distinct lamellated or “onionskin” appearance characterized by alternating concentric layers of low signal intensity on T2-weighted images. Epidermoid cysts generally show no enhancement after contrast and lack diffusion restriction, with ADC values equal to or greater than normal testicular parenchyma. Recognizing this distinctive pattern allows accurate preoperative diagnosis, thus facilitating conservative management and avoiding unnecessary radical orchiectomy [46].

#### 3.3.2. Paratesticular and Extratesticular Masses

Paratesticular and extratesticular masses comprise a diverse group of lesions that arise from the epididymis, spermatic cord, tunica vaginalis, and surrounding soft tissues. Most paratesticular lesions are benign (about 75%), so distinguishing them from intratesticular masses before surgery is important to prevent unnecessary orchiectomy [1,7,8]. US remains the first-line modality due to its availability and excellent spatial resolution; however, it can be limited in evaluating the exact tissue origin, internal composition, and extent of certain complex or large lesions [47].

Among the most common benign paratesticular masses are lipomas, adenomatoid tumors, fibrous pseudotumors, and leiomyomas. Lipomas are the most frequent benign tumors of the spermatic cord and typically show high T1- and T2-weighted signal intensity that suppresses on fat-saturated sequences, with no internal enhancement, which helps distinguish them from liposarcomas [15,39]. Adenomatoid tumors are the most common benign tumors of the epididymis, often presenting as small, well-defined, hypovascular lesions with low to intermediate T2 signal and minimal post-contrast enhancement. MRI helps confirm their extratesticular origin and rule out invasion of the testicular parenchyma [39,47].

Fibrous pseudotumors are reactive benign proliferations of the tunica vaginalis or albuginea, usually secondary to prior trauma or inflammation. On MRI, they present as well-defined masses with low T2 signal due to their fibrous content and may enhance mildly post-contrast; this pattern can help avoid misdiagnosis as malignancy. Leiomyomas are less common but may arise from smooth muscle within the epididymis or tunica, appearing as well-circumscribed, solid masses with intermediate T1 and low T2 signal and variable enhancement [8,48].

Differentiating benign from malignant extratesticular tumors is critical, as malignant lesions such as rhabdomyosarcomas, liposarcomas, leiomyosarcomas, and metastatic deposits, although rare, require aggressive management. Malignant paratesticular tumors typically present as large, lobulated masses with heterogeneous T2 signal, necrosis, irregular margins, and more avid enhancement compared to benign counterparts. Metastases to the spermatic cord or epididymis are rare but can occur from primary tumors of the prostate, kidney, gastrointestinal tract, or lymphoma [48,49,50].

### 3.4. Infertility

Male infertility is a heterogeneous condition with multiple potential causes, and its initial evaluation relies overwhelmingly on clinical assessment, hormonal profiling, and detailed semen analysis. These clinical and laboratory parameters—such as semen volume, sperm concentration and motility, endocrine status, and biochemical markers of obstruction form the basis of diagnostic decision-making. The role of imaging in this workup is therefore limited and reserved for selected situations in which structural abnormalities are suspected after the primary evaluation has been completed [51,52].

Azoospermia, defined as the complete absence of spermatozoa in the ejaculate, affects approximately 10–20% of infertile men and is categorized as obstructive (OA) or non-obstructive azoospermia (NOA). Distinguishing between these entities is crucial, as OA is amenable to microsurgical correction or sperm retrieval, while NOA, related to testicular failure, typically requires testicular sperm extraction (TESE) for use in assisted reproduction [53].

While scrotal ultrasonography is the primary imaging tool, MRI can be invaluable when the findings are equivocal. T2-weighted sequences allow precise evaluation of testicular volume, parenchymal signal, and epididymal anatomy. DWI adds functional data on tissue integrity. In a prospective study, ADC values were significantly higher in NOA (mean 1.114 × 10^−3^ mm^2^/s) compared to OA (mean 0.876 × 10^−3^ mm^2^/s), likely reflecting loss of germinal elements and increased interstitial space. A threshold of ≥0.952 × 10^−3^ mm^2^/s provided 81% sensitivity and 90% specificity for NOA diagnosis [54].

MRI is increasingly studied as a non-invasive tool to predict the presence of spermatozoa in NOA patients prior to TESE. ADC values are elevated in testes with Sertoli cell-only syndrome (SCOS) and maturation arrest compared to hypospermatogenesis. A negative correlation has been demonstrated between ADC and histological severity. Magnetization transfer ratio (MTR), reflecting macromolecular content, is also increased in NOA and correlates inversely with spermatogenesis [55].

Unlike traditional ROI-based methods, volumetric ADC analysis evaluates the entire testicular volume. In a recent study, metrics such as the 25th percentile and median ADC differentiated histologic subtypes. Median ADC was the strongest predictor of sperm retrieval, with statistically significant correlation (*p* = 0.007) [56].

Diffusion tensor imaging (DTI) provides additional insight into the anisotropy of testicular microstructure. In NOA, fractional anisotropy (FA) is paradoxically increased, especially in SCOS, possibly due to simplification and rigidity of the seminiferous architecture. In men with NOA who experience negative TESE outcomes, visual analysis of fiber tracts often reveals abnormalities, including shortened, disrupted, or disorganized configurations [57].

Proton spectroscopy enables the in vivo assessment of metabolic activity in testicular tissue. In NOA, reduced levels of choline, creatine, and myo-inositol are consistently observed. In one pilot study, choline ≥ 1.46 ppm and creatine ≥ 1.43 ppm was associated with positive TESE outcomes, while NOA patients with prior failed TESE had significantly lower metabolite signals [58].

Currently, MRI is not part of standard infertility work-up, but its role is expanding. The European Society of Urogenital Radiology recognizes MRI’s potential for lesion characterization and undescended testis assessment, and early studies suggest it may become a valuable adjunct in evaluating NOA and selecting candidates for TESE [59]. Functional MRI metrics (ADC, MTR, FA, spectroscopy) show promise as non-invasive biomarkers of spermatogenic activity (Table 3), although broader validation is needed before routine implementation.

## 4. Penile Pathology

### 4.1. Penile Neoplasms

Penile cancer is a rare malignancy, most commonly squamous cell carcinoma (SCC), accounting for over 95% of cases. Accurate local staging is critical to guide treatment decisions, particularly in determining the feasibility of organ-sparing surgery versus partial or total penectomy. While physical examination and ultrasound are useful for initial assessment, MRI is the preferred modality for precise T-staging and evaluating the extent of deep tissue invasion [6].

The 8th edition of the American Joint Committee on Cancer (AJCC) TNM staging system introduced notable changes emphasizing the role of MRI in accurate local tumor assessment. According to this classification, tumor invasion into the corpus spongiosum is designated as T2, whereas invasion into the corpus cavernosum (including the tunica albuginea) is classified as T3 disease. In contrast, urethral involvement, previously categorized separately, is no longer staged independently, although recognizing urethral infiltration remains clinically relevant for surgical decision-making [13,60].

**Figure 7:**
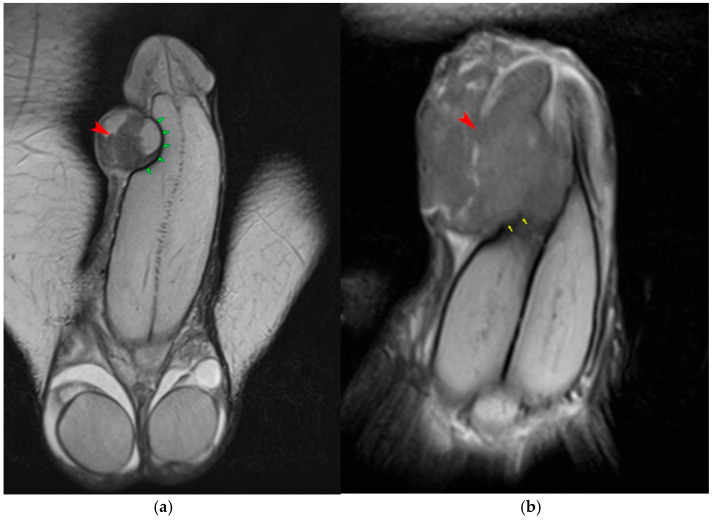
Squamous cell carcinoma of the penis in two different patients. (**a**) Coronal T2-weighted image shows a penile mass (red arrowhead) without invasion of the corpora cavernosa; the tunica albuginea (green arrows) remains intact. (**b**) In another patient, the tumor (red arrowhead) infiltrates the corpora cavernosa, evidenced by disruption of the tunica albuginea (yellow arrows).

MRI is particularly valuable in differentiating between T2 (corpus spongiosum involvement) and T3 (corpora cavernosa involvement) tumors, a distinction challenging to ascertain accurately by clinical examination alone. High-resolution T2-weighted sequences, which demonstrate the hypointense tunica albuginea and hyperintense corpora cavernosa and spongiosum, significantly enhance the assessment of tumor depth and local extent (Figure 7). Multiparametric MRI, combining anatomical imaging with DWI and dynamic contrast-enhanced sequences, further improves diagnostic confidence. DWI helps identify tumor cellularity, while DCE sequences delineate tumor vascularity and accurately define tumor boundaries [61,62]. Recent studies have shown the excellent concordance between mpMRI and histopathological findings regarding tumor localization, size, infiltration depth, and T-staging accuracy, without the need for pharmacologically induced penile erection [63,64].

Despite the benefits, MRI does possess limitations. It may be less sensitive for early or subtle urethral involvement and in differentiating fibrosis from residual tumor tissue following prior biopsies or treatments [62,65]. Moreover, while MRI can detect enlarged regional lymph nodes, it cannot reliably differentiate reactive nodes from metastatic nodes, and therefore surgical staging remains the definitive approach in high-risk cases [13,62].

Current guidelines from the EAU now recommend MRI for preoperative local staging of penile cancer, especially when organ-preserving surgeries are considered. This recommendation is based upon MRI’s proven effectiveness in defining precise tumor anatomy and enabling accurate patient selection for conservative management, thereby potentially reducing recurrence and improving patient outcomes [60].

**Figure 8:**
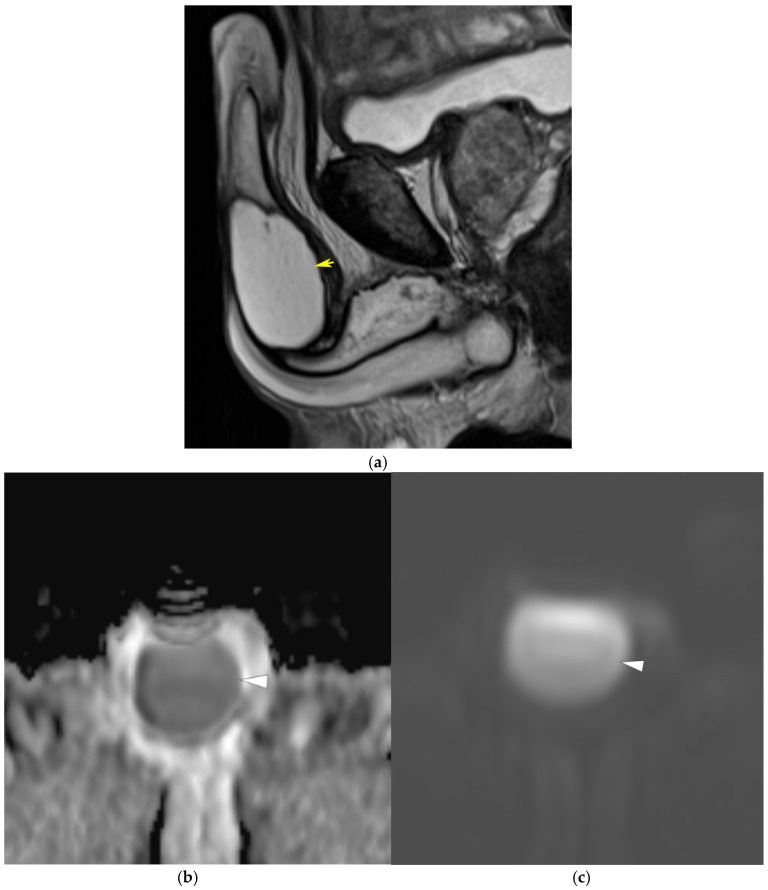
Penile abscess. (**a**) Sagittal T2-weighted image demonstrates a fluid-filled mass in the mid-portion of the penis (yellow arrow). (**b**) Corresponding apparent diffusion coefficient (ADC) map and (**c**) diffusion-weighted image reveal marked diffusion restriction within the lesion (white arrowhead), consistent with abscess formation.

Although penile cancer is the most significant malignant lesion requiring precise staging, a range of non-neoplastic and benign conditions may present with overlapping clinical or imaging features. Abscesses, for instance, can manifest as painful, irregularly enhancing masses, but MRI typically shows central fluid signal with markedly restricted diffusion, peripheral rim enhancement and adjacent inflammatory changes, in contrast to the solid, low-to-intermediate T2 signal intensity and more homogeneous enhancement of squamous cell carcinoma (Figure 8). Furthermore, although rare, benign penile tumors such as leiomyomas, hemangiomas, lipomas, and neurofibromas may also enter the differential diagnosis. These lesions typically appear well circumscribed and lack deep cavernosal invasion, with signal characteristics reflecting their underlying tissue composition. For example, hemangiomas are hyperintense on T2-weighted images with vivid post-contrast enhancement; lipomas follow fat signal intensity with complete suppression on fat-saturated sequences; and leiomyomas demonstrate low-to-intermediate T2 signal. Awareness of these mimics and careful interpretation with multiparametric MRI are therefore essential to avoid unnecessary radical surgery and guide appropriate management [14].

### 4.2. Penile Trauma

Penile trauma is a urological emergency typically caused by blunt injury to an erect penis, most commonly during sexual intercourse. This injury usually results in a penile fracture, defined as a rupture of the tunica albuginea surrounding the corpora cavernosa, often accompanied by a characteristic clinical presentation including a cracking sound, immediate detumescence, severe pain, swelling, and ecchymosis. Although diagnosis is often made clinically, MRI may be helpful when the diagnosis is uncertain or when the extent of injury requires precise definition for surgical planning [6,66].

MRI findings in penile fracture include a discontinuity or rupture of the hypointense tunica albuginea clearly identified on T2-weighted images, frequently associated with intracavernosal or extratunical hematoma formation. The signal intensity of the hematoma varies depending on the age of the blood products; acute hematomas are usually hyperintense on T2-weighted images, whereas subacute hematomas become hyperintense on T1-weighted sequences due to methemoglobin presence. The presence and extent of these hematomas provide vital clues for evaluating the severity and anatomical details of the injury, significantly aiding the surgeon in precise surgical exploration and repair [66].

MRI is particularly useful in cases with atypical presentations or equivocal clinical findings, such as those with suspected proximal penile injury involving the crus or suspensory ligament, which might be difficult to assess clinically or with ultrasound alone [23]. In addition, MRI provides superior visualization of associated injuries, including involvement of the corpus spongiosum or the penile urethra, the latter being critical because missed urethral injury can lead to severe long-term complications. In suspected urethral involvement, dedicated sagittal and axial T2-weighted images, and post-contrast T1-weighted sequences clearly depict discontinuity or irregularities of the urethral wall, although traditional retrograde urethrography remains the standard of care [67].

In addition to conventional sequences, the use of post-contrast imaging may further clarify the exact location and extent of the rupture, particularly in complex or ambiguous cases. MRI also allows the identification of additional traumatic sequelae, such as fibrosis, abscess formation, or fistulae, which can impact the clinical management approach significantly [23,66].

### 4.3. Priapism

Priapism is a prolonged penile erection lasting more than four hours and is classified into ischemic (low-flow), non-ischemic (high-flow), and stuttering subtypes.

Ischemic priapism accounts for most cases and constitutes a urologic emergency because sustained hypoxia can precipitate corporal fibrosis and irreversible erectile dysfunction. MRI provides valuable diagnostic and prognostic information in this context, particularly by assessing tissue viability to guide urgent management decisions [6,68]. In ischemic priapism, stagnant, deoxygenated blood leads to diffuse or segmental T1 hyperintensity within the corpora cavernosa from methemoglobin, with corresponding T2 hyperintensity reflecting edema and hemorrhagic necrosis; after contrast, non-viable tissue shows decreased or absent enhancement, a finding that predicts irreversible injury and supports consideration of early penile prosthesis placement [69]. In contrast, non-ischemic priapism typically follows perineal trauma with formation of an arteriocavernosal fistula; MRI may show focal early enhancement on dynamic sequences and delineate a discrete pseudoaneurysm, which can be further characterized with time-resolved contrast-enhanced MR angiography or Doppler ultrasound [6,68].

MRI is particularly useful when ischemic priapism has persisted beyond 48–72 h and corporal viability is uncertain, for preoperative mapping of the extent and distribution of irreversible damage, when history and ultrasound are inconclusive in distinguishing ischemic from non-ischemic etiologies, and for evaluating complications such as fibrosis, pseudoaneurysm, or persistent fistulous tracts [6,69].

### 4.4. Peyronie’s Disease

Peyronie’s disease is a connective tissue disorder of the penis characterized by the formation of fibrous plaques within the tunica albuginea, commonly presenting clinically as penile curvature, pain during erection, and sexual dysfunction. This condition typically affects middle-aged and older men, causing substantial impairment in sexual health and quality of life. Although clinical examination and ultrasonography are commonly utilized for initial diagnosis, MRI provides unique advantages for a detailed evaluation of plaque characteristics and their precise anatomical extent, particularly in complex cases or when surgical treatment is contemplated [70,71,72,73].

**Figure 9:**
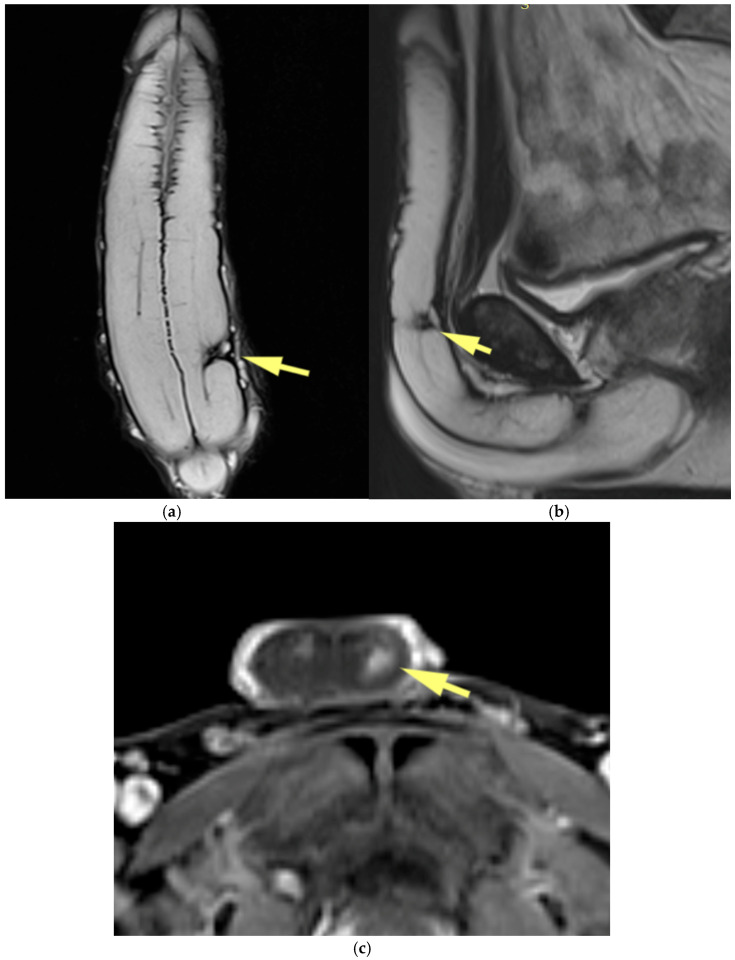
Peyronie’s disease. (**a**) Coronal and (**b**) sagittal T2-weighted images show a hypointense plaque (yellow arrow) in the mid-portion of the left corpus cavernosum. (**c**) Axial contrast-enhanced T1-weighted image demonstrates avid enhancement of the plaque (yellow arrow), indicating active disease.

On MRI, the appearance of Peyronie’s plaques varies according to the disease stage. Early or active plaques, characterized by inflammation and edema, usually present as areas of mild hyperintensity on T2-weighted images, reflecting increased water content. These active plaques also typically demonstrate enhancement after intravenous gadolinium administration due to their inflammatory vascularity. In contrast, chronic or mature plaques are composed predominantly of dense fibrous tissue and sometimes calcification; thus, they appear hypointense on both T1- and T2-weighted sequences, with little or no contrast enhancement, highlighting their inactive, stable nature [6,74] (Figure 9.).

MRI is particularly beneficial in demonstrating subtle, deep, or non-palpable plaques that may escape clinical or ultrasonographic detection. Plaques typically involve the dorsal or dorsolateral aspects of the penile shaft but can extend deeply toward the intercavernosal septum or urethra. Such deep extensions are often difficult to assess with clinical examination alone, making MRI especially valuable in preoperative planning by providing accurate anatomical delineation of plaque location, depth, and relationship to adjacent structures [70,71].

Moreover, MRI with artificial erection, induced pharmacologically by intracavernosal injection of prostoglandin, can directly visualize penile curvature, helping surgeons precisely assess the degree and direction of penile deformity. This information significantly enhances surgical planning, improving outcomes in patients undergoing plaque incision, grafting procedures, or penile prosthesis implantation [6,70].

MRI findings can also be employed to guide medical treatment decisions. The presence of active inflammatory plaques with MRI enhancement may indicate disease activity, suggesting that conservative or medical therapy might be effective. Conversely, stable fibrotic plaques without significant enhancement typically indicate a chronic stage, where surgical correction becomes more appropriate [70,74].

### 4.5. Penile Prostheses

Penile prostheses represent an important therapeutic option for men with refractory erectile dysfunction, commonly arising secondary to conditions such as severe Peyronie’s disease, ischemic priapism, or postoperative complications after radical prostatectomy. The most frequently implanted devices are inflatable prostheses, comprising intracavernosal cylinders, a scrotal pump, and an abdominal fluid reservoir, and malleable (semi-rigid) prostheses, consisting of paired rods placed within the corpora cavernosa [6].

While clinical examination and ultrasonography usually provide sufficient initial evaluation for penile prosthesis complications, MRI becomes invaluable in complex or uncertain scenarios. MRI is especially beneficial in situations where prosthesis malfunction, malposition, erosion, or postoperative infection is suspected, and when clinical findings are unclear or contradictory [6,75].

MRI of penile prostheses poses specific technical challenges due to potential artifacts produced by the implanted material, particularly older or mixed-material prostheses containing metallic components. However, newer-generation penile prostheses constructed from MRI-compatible materials such as silicone or polyurethane demonstrate significantly fewer artifacts, allowing reliable diagnostic assessment [6,76].

MRI effectively assesses mechanical failures such as fluid leaks, cylinder disruptions, or tubing disconnections. These abnormalities manifest as structural discontinuities or contour irregularities on high-resolution T2-weighted imaging. In particular, for inflatable devices, MRI performed with dynamic imaging in both inflated and deflated states can further assist in diagnosing subtle mechanical failures or incomplete inflation, greatly aiding surgical planning. Malleable prostheses appear as uniformly hypointense structures across all MRI sequences, and their correct placement or malposition can be evaluated by examining the surrounding penile tissue and corporal boundaries [77,78].

MRI is also highly sensitive in detecting complications related to prosthesis infection, which typically appear as fluid collections or abscess formation with peripheral enhancement after intravenous contrast administration. Enhanced soft tissue reaction around the prosthesis and fistulous tracts extending to the penile surface or surrounding tissues can be accurately characterized, significantly impacting clinical decision-making and potentially guiding prosthetic revision surgery [3,6].

## 5. Discussion

Magnetic resonance imaging of the scrotum and penis has evolved considerably since 2020. In scrotal pathology, prospective studies have validated multiparametric MRI protocols for characterizing sonographically indeterminate testicular masses and identifying candidates for testis-sparing surgery, using combined morphologic, DWI, and DCE criteria [1,33,37].

In male infertility, testicular MRI has progressed from descriptive assessments of testicular size and signal to quantitative biomarkers such as ADC histogram metrics, magnetization transfer ratio, and diffusion tensor parameters, which correlate with spermatogenic status and TESE outcomes. These advances suggest MRI may eventually assist in non-invasive selection of candidates for surgical sperm retrieval, although this remains outside routine work-up and is not yet recommended by guidelines [56,59].

In penile oncology, recent data demonstrate that non-erect mpMRI without pharmacologic erection can accurately assess corpus spongiosum and cavernosum invasion and shows high concordance with histopathology, supporting its use for T2/T3 discrimination and selection for organ-preserving surgery. These studies complement updated EAU–ASCO recommendations that now explicitly recognize MRI as a key tool for local staging when conservative surgery is contemplated [60,64].

Despite these advances, several unresolved gaps and controversies remain. The most prominent is the limited role of MRI in the acute scrotum. Although MRI demonstrates excellent performance for diagnosing segmental testicular infarction, traumatic rupture, and even torsion, real-world emergency workflows rarely permit its use. In suspected torsion, MRI is explicitly discouraged by both urological and radiological guidelines because even short delays may compromise testicular viability, ultrasound remains the only feasible imaging tool in time-sensitive presentations. Similarly, while MRI can accurately differentiate abscess from tumor, detect subtle tunical disruptions, and map the spread of necrotizing infections, these benefits generally apply to subacute or equivocal cases rather than true emergencies [5,79].

Another major limitation lies in inter-reader variability. Few studies have systematically evaluated agreement among radiologists interpreting scrotal or penile MRI, but available evidence shows variability in assessing tunica albuginea invasion, subtle enhancement patterns, and the distinction between fibrosis and viable tumor particularly following biopsy or prior treatment. This inconsistency underscores the need for standardized reporting systems similar to those used in prostate or breast MRI, which currently do not exist for male genital imaging [4,64,80].

Accessibility, cost, and workflow barriers further limit widespread adoption. High-quality penile and scrotal MRI requires meticulous technique, patient cooperation, and motion control, and is susceptible to artifact from metallic prostheses, prior surgeries, or patient movement. Furthermore, MRI remains unavailable in many emergency settings, and when available, requires longer acquisition and interpretation times compared to ultrasound. These realities explain the continued reliance on ultrasound as the primary imaging modality, with MRI serving only in situations where additional specificity is essential for guiding management [3,12,15].

Moreover, although MRI adds substantial anatomical detail, it is important to recognize scenarios where MRI findings do not meaningfully alter management. In testicular cancer, radical orchiectomy remains the standard of care for solid intratesticular masses, regardless of MRI subtype differentiation. Similarly, in penile cancer, MRI cannot reliably differentiate reactive from metastatic lymphadenopathy, and nodal staging continues to rely on surgical assessment [30,81].

MRI has no meaningful role in the evaluation of varicocele or erectile dysfunction, where Doppler ultrasonography is clearly superior. Varicocele diagnosis depends on real-time assessment of venous reflux, flow direction, and hemodynamic changes during Valsalva, none of which MRI can reliably capture. Similarly, erectile dysfunction requires evaluation of cavernosal arterial inflow and veno-occlusive competence parameters inherently dependent on dynamic Doppler techniques. Although MRI can show structural abnormalities such as fibrosis or plaques, these findings rarely modify management and do not justify MRI as part of routine diagnostic evaluation [52,82].

## 6. Conclusions

Magnetic resonance imaging has matured into a decisive complement to ultrasonography across the spectrum of scrotal and penile disease. When applied with appropriate indications and optimized technique, mpMRI provides high-resolution anatomic detail together with functional information that clarifies equivocal ultrasound findings, refines differential diagnoses, and maps disease extent with surgical precision. In acute settings, MRI helps characterize trauma and delineate infectious complications; in oncologic pathways, it improves localization and local staging of testicular and penile tumors and can support organ-preserving strategies. In andrology, emerging quantitative metrics show promise as non-invasive markers of spermatogenic status and may assist in selecting candidates for TESE. For priapism and Peyronie’s disease, MRI informs timing and type of intervention by distinguishing viable from non-viable tissue and by precisely mapping plaque burden and activity. In the setting of penile prostheses, it serves as a problem-solving tool to detect malposition, mechanical failure, or infection when clinical assessment and sonography are inconclusive.

Broader adoption now hinges on standardizing protocols, reporting the key descriptors that drive management (lesion origin, tunical and adjacent structure involvement, diffusion and enhancement behavior, and relevant quantitative metrics), and strengthening collaboration between radiologists and urologists. While MRI has limitations, including availability, cost, motion and artifact sensitivity, and the need for specialized interpretation, these must be balanced against its ability to prevent unnecessary surgery, guide timely intervention, and improve patient outcomes.

Future work should prioritize multicenter prospective studies, harmonized mpMRI protocols, and histopathologic correlation to validate quantitative thresholds. Integration of radiomics and AI may further enhance lesion characterization (particularly for small, incidentally detected testicular masses) and improve prediction of clinically meaningful endpoints such as sperm retrieval in NOA or candidacy for organ-preserving oncologic surgery. Taken together, the evidence supports MRI as an important, patient-centered imaging modality in the multidisciplinary care of male genital pathologies.

## Figures and Tables

**Table 1:** MRI features of acute scrotal pathology.

Condition	Key MRI Findings	DWI/Enhancement Characteristics	Clinical Implications
Testicular torsion	Enlarged, hypointense testis; twisted cord (‘whirlpool sign’)	Absent enhancement; diffusion restriction	Viability assessment
Trauma/rupture	Discontinuity of tunica albuginea; heterogeneous signal	Non-enhancing hematoma	Guides exploration/conservative follow-up
Orchitis/epididymo-orchitis	T2 hyperintense, enlarged testis/epididymis; wall thickening	Diffuse enhancement	Distinguish from tumor; assess abscess
Abscess	T2 hyperintense core, rim enhancement	Restricted diffusion	Differentiates abscess from necrotic tumor
Fournier gangrene	Fascial thickening, fluid tracking, non-enhancing necrosis	Variable	Mapping for debridement

**Table 2:** MRI features of common testicular tumors.

Tumor Type	T2 Signal	Enhancement	ADC (×10^−3^ mm^2^/s)	Key Features
Seminoma	Homogeneous high	Uniform	1.0–1.2	Lobulated, solid
NSGCT	Heterogeneous	Irregular	0.7–0.9	Cystic/necrotic components
Leydig cell tumor	Iso–mild high	Early intense	~1.2	Small, sharply defined
Epidermoid cyst	Lamellated low	None	No restriction	Onion-skin appearance

**Table 3:** MRI biomarkers in male infertility.

Condition	Testis Volume	ADC (×10^−3^ mm^2^/s)	MRS: Choline (ppm)	Prognosis
Obstructive azoospermia	Normal	<1.0	Normal	Excellent
Non-obstructive azoospermia	Small	>1.1	↓ Cho, Cr	Variable
Hypospermatogenesis	Normal	1.0–1.2	↑ Cho	Favorable

## Data Availability

No new data were created or analyzed in this study. Data sharing is not applicable to this article.

## Abbreviations

ADC	Apparent Diffusion Coefficient
AJCC	American Joint Committee on Cancer
AUA	American Urological Association
BCD	Bell Clapper Deformity
Cho	Choline
Cr	Creatine
DCE	Dynamic Contrast-Enhanced
DTI	Diffusion Tensor Imaging
DWI	Diffusion-Weighted Imaging
EAU	European Association of Urology
ESUR	European Society of Urogenital Radiology
FA	Fractional Anisotropy
FOV	Field of View
GCT	Germ Cell Tumor
LCT	Leydig Cell Tumor
MRS	Magnetic Resonance Spectroscopy
MRI	Magnetic Resonance Imaging
mpMRI	Multiparametric Magnetic Resonance Imaging
MTR	Magnetization Transfer Ratio
NOA	Non-Obstructive Azoospermia
NSGCT	Nonseminomatous Germ Cell Tumor
OA	Obstructive Azoospermia
ppm	Parts per Million
SCOS	Sertoli Cell-Only Syndrome
SCC	Squamous Cell Carcinoma
SCST	Sex Cord-Stromal Tumor
TESE	Testicular Sperm Extraction
TGCT	Testicular Germ Cell Tumor
TSE	Turbo Spin Echo
US	Ultrasonography
